# Neutrophil Extracellular Trap Components Associate with Infarct Size, Ventricular Function, and Clinical Outcome in STEMI

**DOI:** 10.1155/2019/7816491

**Published:** 2019-10-21

**Authors:** Ragnhild Helseth, Christian Shetelig, Geir Øystein Andersen, Miriam Sjåstad Langseth, Shanmuganathan Limalanathan, Trine B. Opstad, Harald Arnesen, Pavel Hoffmann, Jan Eritsland, Ingebjørg Seljeflot

**Affiliations:** ^1^Center for Clinical Heart Research, Oslo University Hospital Ullevål, Norway; ^2^Department of Cardiology, Oslo University Hospital Ullevål, Norway; ^3^Department of Internal Medicine, Drammen Hospital, Vestre Viken Hospital Trust, Norway; ^4^University of Oslo, Norway; ^5^The National Association for Heart and Lung Disease (LHL) Hospital, Gardermoen, Norway

## Abstract

**Background:**

The relevance of neutrophil extracellular traps (NETs) in acute ST-elevation myocardial infarction (STEMI) is unclear. We explored the temporal profile of circulating NET markers and their associations to myocardial injury and function and to adverse clinical events in STEMI patients.

**Methods and Results:**

In 259 patients, blood samples were drawn before and after PCI, on day 1, and after 4 months. Double-stranded deoxyribonucleic acid (dsDNA) and myeloperoxidase-DNA (MPO-DNA) were measured in serum by a nucleic acid stain and ELISA. Cardiac magnetic resonance imaging assessed microvascular obstruction (MVO), area at risk, infarct size, myocardial salvage index, left ventricular ejection fraction (LVEF), and change in indexed left ventricular end-diastolic volume (LVEDVi). Clinical events were registered after 12 months. dsDNA and MPO-DNA levels were highest before PCI, with reduced levels thereafter (all *p* ≤ 0.02). Patients with high vs. low day 1 dsDNA levels (>median; 366 ng/ml) more frequently had MVO, larger area at risk, larger infarct size acutely and after 4 months, and lower myocardial salvage index (all *p* < 0.03). Moreover, they had lower LVEF acutely and after 4 months, and larger change in LVEDVi (all *p* ≤ 0.014). High day 1 dsDNA levels also associated with risk of having a large infarct size (>75th percentile) and low LVEF (≤49%) after 4 months when adjusted for gender, time from symptoms to PCI, and infarct localization (OR 2.3 and 3.0, both *p* < 0.021), and patients with high day 1 dsDNA levels were more likely to experience an adverse clinical event, also when adjusting for peak troponin T (hazard ratio 5.1, *p* = 0.012). No such observations were encountered for MPO-DNA.

**Conclusions:**

High day 1 dsDNA levels after STEMI were associated with myocardial infarct size, adverse left ventricular remodeling, and clinical outcome. Although the origin of dsDNA could be discussed, these observations indicate a potential role for dsDNA in acute myocardial ischemia. This trial is registered with S-08421d, 2008/10614 (Regional Committee for Medical Research Ethics in South-East Norway (2008)).

## 1. Introduction

Despite today's state-of-the-art management of acute ST-elevation myocardial infarction (STEMI), including rapid revascularization with percutaneous coronary intervention (PCI) and modern antithrombotic treatment, one-year mortality still remains approximately 10% [1]. While the inflammatory aspect of atherosclerosis and coronary artery disease (CAD) development is well established [2], data also indicate that the inflammatory response to acute myocardial ischemia affects infarct size and how the left ventricle is remodeled [3, 4]. The delicate interplay and transition from the early post-STEMI proinflammatory phase where myocardial ischemia and cell death lead to the production of reactive oxygen species (ROS), the recruitment of immune cells, and a general proinflammatory “cytokine burst” to the subsequent reparative phase aimed at myocardial healing and scar formation, are complex and poorly understood [3, 4].

As first-line defenders against injury, circulating neutrophils infiltrate ischemic myocardium within hours of injury [3]. Neutrophils are key effectors in the early postinfarction proinflammatory phase by phagocytosing cellular debris, generating ROS, degrading extracellular matrix through neutrophil granule proteins, and chemotaxis involving monocytes [3–5]. Epidemiological data reporting that increased neutrophil count after PCI in STEMI patients associates with larger infarct size and deteriorated left ventricular function underpin the clinical importance of neutrophil actions in the early postinfarction phase [6].

Recently, it has been shown that neutrophils are able to release web-like structures comprising nuclear chromatin in the form of double-stranded deoxyribonucleic acid (dsDNA) and histones studded with neutrophil proteins into the extracellular space in a process termed NETosis [7]. Neutrophil extracellular traps (NETs) have gained attention in STEMI as they have been identified in coronary thrombi [8–10] and have prothrombotic properties including activation of platelets and the coagulation cascade [11, 12]. A certain association towards infarct size has been reported for circulating dsDNA in STEMI patients [13, 14], whereas NET burden within coronary thrombi has been shown to impact infarct size and ST-segment resolution, the latter representing an indirect measure of coronary no-reflow and ischemia reperfusion (IR) injury [8]. To what extent circulating NET markers in the acute phase of STEMI affect direct IR injury indices, as well as the post-MI left ventricular remodeling process and clinical outcome is unclear. Any contribution of NET components to myocardial injury and function or clinical outcome may pave the way for novel treatment strategies aimed at NET destruction or inhibition.

The aims of this study were, in a cohort of STEMI patients, to explore the temporal profile of the circulating NET markers dsDNA and myeloperoxidase-DNA (MPO-DNA) during the acute event and in a stable condition after 4 months. Whether the NET markers were associated with indices of myocardial injury, left ventricular function, and remodeling assessed by cardiac magnetic resonance (CMR), or with adverse clinical outcome, were further investigated. As the specificity of circulating dsDNA as a NET marker beyond a marker of cellular death can be questioned, gene expression of peptidylarginine deiminase 4 (PAD4), an assumed essential enzyme for NETosis, was also measured in circulating leukocytes in a subset of the cohort.

## 2. Material and Methods

This study was a substudy of the POSTEMI (Postconditioning in ST-elevation Myocardial Infarction) trial, a prospective, randomized, single-center nonblinded clinical trial aimed at investigating whether ischemic postconditioning has cardioprotective effects [15]. As previously reported, no cardioprotective effects on infarct size or other prespecified study outcomes were observed [16]. The POSTEMI trial was approved by the Regional Committee for Medical Research Ethics in South-East Norway in 2008 (registration number S-08421d, 2008/10614), and all included patients gave written informed consent. The study conformed to the principles outlined in the Declaration of Helsinki. The supporting CONSORT (CONsolidated Standards Of Reporting Trials) checklist is provided in the Supplementary Materials. In brief, 272 patients with first-time STEMI admitted to Oslo University Hospital Ullevål within 6 hours of symptom onset were included between January 2009 and August 2012. Patients were randomized in a 1 : 1 fashion to ischemic postconditioning or standard care after angiographic verification of an acute coronary occlusion (Thrombolysis In Myocardial Infarction (TIMI) 0-1 flow) and successful revascularization (TIMI 2-3 flow) of the infarct-related artery (IRA). Patients with previous myocardial infarction (MI), renal failure, contraindications to CMR, clinical instability, or who were unable to give informed consent, were excluded.

### 2.1. Laboratory Analyses

Blood samples were drawn before and immediately after PCI, at day 1, and after 4 months. The median time from symptom onset to blood sampling before PCI was 2.8 hours, while the day 1 blood samples were drawn at a median of 18.3 hours after PCI. Serum was separated within 1 hour by centrifugation at 2500 *g* for 10 min and kept frozen at -80°C until analyzed for dsDNA and myeloperoxidase-deoxyribonucleic acid (MPO-DNA) in batches.

Levels of dsDNA were quantified by the fluorescent nucleic acid stain Quant-iT PicoGreen® (Invitrogen Ltd., Paisley, UK) and fluorometry (Fluoroskan Ascent® fluorometer, Thermo Fisher Scientific Oy, Vantaa, Finland). Levels of MPO-DNA were quantified by an in-house enzyme-linked immunosorbent assay (ELISA) technique [17] where plates were coated and incubated overnight with the capture antibody anti-MPO (AbD Serotec, Hercules, CA, USA) and, after blocking with bovine serum albumin (BSA), patient serum and a peroxidase-labeled anti-DNA antibody (Cell Death Detection Kit, Roche Diagnostics GmbH, Mannheim, Germany) were added. After 2 hours of incubation, a peroxidase substrate was added and absorbance was measured after 40 minutes as optical density (OD) units. The interassay CVs for dsDNA and MPO-DNA were 6.1% and 10.3%, respectively.

For gene expression of PAD4, PAXgene Blood RNA tubes collected immediately after PCI and on day 1 were used for RNA extraction from circulating leukocytes in a subset of 100 consecutively included patients. Total RNA was reversely transcribed into complementary DNA (cDNA) by the use of qScript cDNA SuperMix (Quanta BioSciences, Inc., Gaithersburg, USA), and expression of PAD4 mRNA was assessed by real-time polymerase chain reaction (RT-PCR) on the ViiA 7 Real-Time PCR System (Applied Biosystems, by Life Technologies, Foster City CA, USA) using TaqMan Universal PCR Master Mix, No AmpErase UNG, and the PAD4 TaqMan assay (Hs01057483_m1). PAD4 mRNA levels were measured as relative quantification (RQ) (2^-ΔΔCt^ method) [18] with beta-2-microglobulin (*β*_2_M) as housekeeping gene (Assay ID Hs99999907_m1) (all Applied Biosystems).

Serial measurements of serum troponin T were performed by electrochemiluminescence technology (Elecsys 2010, Roche Diagnostics GmbH, Mannheim, Germany), and levels of N-terminal pro-B-type natriuretic peptide (NT-proBNP) were determined by an Elecsys proBNP sandwich immunoassay on an Elecsys 2010. C-reactive protein (CRP) was measured by conventional routine laboratory methods.

### 2.2. CMR Analyses

Details regarding the CMR protocol in the POSTEMI trial have been published previously [19]. In brief, CMR was performed at a median of 2 days after admission and repeated after 4 months. All images were taken with a 1.5 T scanner (Philips Intera, release 11 or Philips Achieva, release 3.2, Philips Medical Systems, Best, The Netherlands). T2-weighted images in the short-axis plane were used for the determination of the “area at risk,” defined as myocardium with a signal intensity of >2 standard deviations above the signal intensity in remote, noninfarcted myocardium. Images with late gadolinium enhancement obtained 15 minutes after contrast injection (gadolinium-DTPA 469 mg/ml, 0.15 mmol/kg; Magnevist, Schering AG, Germany) in short-axis and 2- and 4-chamber long-axis views were used to calculate infarct size. Microvascular obstruction (MVO), defined as a dark area within the hyperintense area of infarcted myocardium, was determined in late enhancement images in the acute phase as present or not. Myocardial salvage index (%) was calculated as [(area at risk in the acute phase − infarct size after 4 months)/area at risk in the acute phase)]×100. LV short-axis images were obtained for volume analyses including left ventricular ejection fraction (LVEF), indexed LV end-systolic volume (LVESVi), and indexed left ventricular end-diastolic volume (LVEDVi).

### 2.3. Adverse Clinical Events and Follow-Up

Adverse clinical events, defined as a composite endpoint of death, MI, unscheduled revascularisation ≥ 3 months after the index infarction, stroke, or rehospitalization for heart failure, were registered after 4 months and one year.

### 2.4. Statistics

Due to a skewed distribution of the NET markers, nonparametric statistics were used throughout. Correlation analyses were performed by Spearman's rho. For comparisons of NET marker levels at different time points, Friedman's test followed by Wilcoxon's signed rank test were used. For group comparisons of two or more continuous variables, the Mann-Whitney *U* test and the Kruskal-Wallis test were performed. Proportional data were compared using chi-square tests, including the Mantel-Haenszel test for linear-by-linear association. Logistic regression analyses were performed for dsDNA levels > median on day 1 with large infarct size (defined as infarct size > 75th percentile) and low LVEF (<49%) as dependent variables. Covariates were entered into the multivariable models based on either clinical relevance, or an association with dsDNA levels on day 1 or the dependent variable with a *p* value of <0.10. Cox's regression was used for the assessment of dsDNA levels > median on day 1 and risk of adverse clinical endpoints. Due to a modest number of endpoints and thus restriction in how many variables could be included into the model, the TIMI risk score, a composite risk score for the estimation of 1-year mortality in STEMI patients [20], was used. No correction for multiple comparisons was performed, as this was an exploratory, hypothesis-generating study. All statistical analyses were performed by IBM SPSS software version 25 (SPSS Inc., Chicago, Illinois).

## 3. Results

### 3.1. Baseline Characteristics

Baseline characteristics stratified according to below/above median dsDNA levels (median 366 ng/ml) on day 1 are shown in Table 1. Patients with high dsDNA levels on day 1 were significantly more often male and had more often anterior MIs, higher peak troponin T, and peak CRP, as well as higher creatinine levels at admission, than patients with low dsDNA levels on day 1 (Table 1). No significant differences in baseline characteristics were encountered for patients with MPO-DNA levels below/above median on day 1 (data not shown).

### 3.2. Temporal Profiles of NET Markers

**Figure 1:**
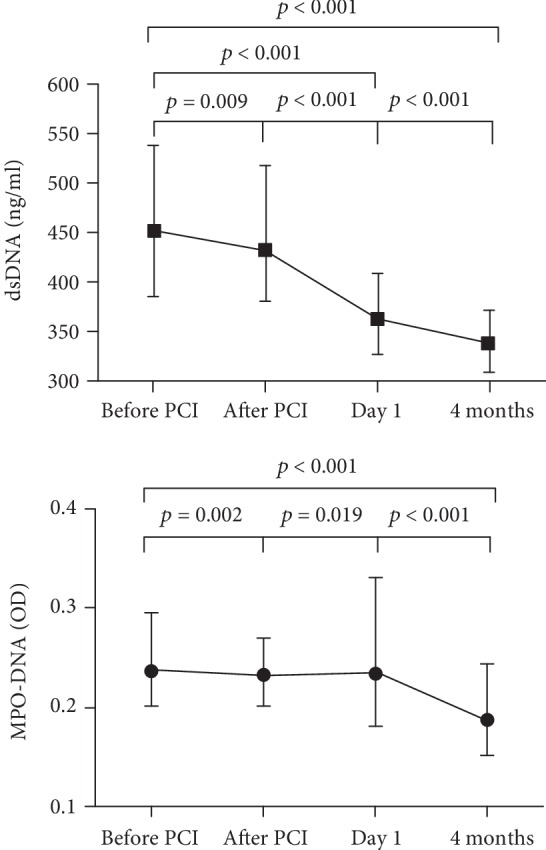
Temporal profiles of NET markers in the total population (*n* = 259 before PCI, *n* = 258 after PCI, *n* = 251/254 for dsDNA/MPO-DNA on day 1, and *n* = 258 at 4 months). Data are presented as median values with 25th and 75th percentiles. dsDNA: double-stranded deoxyribonucleic acid. MPO-DNA: myeloperoxidase-deoxyribonucleic acid. PCI: percutaneous coronary intervention. Friedman's test, Wilcoxon's signed rank test.

Levels of dsDNA and MPO-DNA throughout the study period are shown in Figure 1. Both markers were highest before PCI. While dsDNA levels were significantly reduced at all subsequent time points (all *p* < 0.01), MPO-DNA levels were significantly reduced after PCI and at 4 months (Figure 1). The ischemic postconditioning procedure did not affect the NET marker levels at any time point (data not shown). The results are therefore presented for the total cohort.

The two NET markers intercorrelated moderately but significantly at all time points (*r* = 0.22‐0.40, *p* < 0.001).

PAD4 mRNA levels were significantly higher after PCI compared to on day 1 (2.33 (1.54, 3.81) vs. 0.95 (0.60, 1.38), *p* < 0.001), but they did not correlate significantly to dsDNA or MPO-DNA levels neither after PCI nor on day 1, nor did PAD4 mRNA differ across NET marker quartiles (data not shown). The same pattern was observed for a change in PAD4 mRNA levels (data not shown).

### 3.3. Associations between NET Markers and Indices of Myocardial Infarct Size

dsDNA levels on day 1 correlated significantly to myocardial area at risk and infarct size measured in the acute phase (*r* = 0.264, *p* < 0.001 and *r* = 0.298, *p* < 0.001, respectively) and correlated inversely to myocardial salvage index and to final infarct size measured after 4 months (*r* = −0.157, *p* = 0.033 and *r* = 0.139, *p* = 0.032, respectively). Day 1 MPO-DNA levels correlated weakly to myocardial area at risk and infarct size in the acute phase (*r* = 0.154, *p* = 0.032 and *r* = 0.137, *p* = 0.041, respectively).

**Figure 2:**
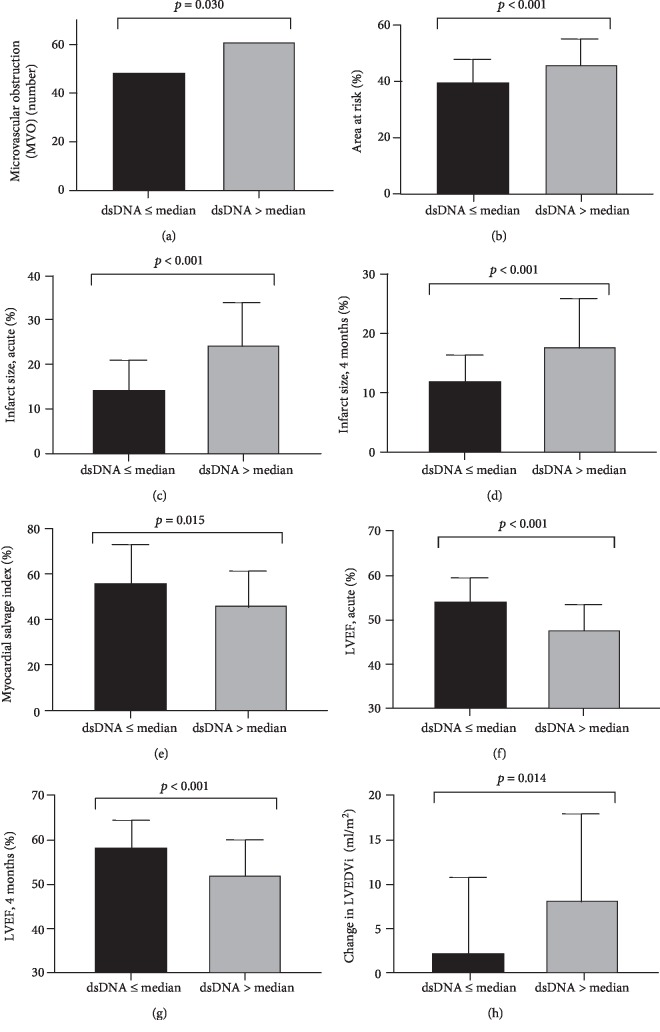
Indices of myocardial infarct size (a, b, c, d, e) and left ventricular remodeling (f, g, h) evaluated by CMR according to circulating levels of dsDNA measured on day 1. (a) *n* = 218, (b) *n* = 192, (c) *n* = 219, (d) *n* = 231, (e) *n* = 185, (f) *n* = 223, (g) *n* = 232, and (h) *n* = 212. LVEF: left ventricular ejection fraction. LVEDVi: left ventricular end-diastolic volume index. dsDNA: double-stranded deoxyribonucleic acid. dsDNA ≤ median: ≤366 ng/ml. dsDNA > median: >367 ng/ml. Chi-square test in (a), Mann-Whitney's test in (b)–(h).

Based on trend analyses of quartiles of dsDNA and MPO-DNA on day 1, the median values were identified as a cut-off for low and high levels related to MVO, area at risk, infarct size, and myocardial salvage index (Supplementary [Supplementary-material supplementary-material-1]). As outlined in Figures 2(a)–2(e), patients with high dsDNA levels on day 1 had significantly more frequent MVO, larger area at risk, larger infarct size in the acute phase and after 4 months, and lower myocardial salvage index (*p* ≤ 0.03 for all). No such relationships were encountered for MPO-DNA (data not shown).

In univariate regression analyses, patients with high dsDNA levels on day 1 had significantly higher risk of having a large final infarct (defined as >75th percentile) at 4-month follow-up (odds ratio (OR): 2.9; 95% confidence interval (CI): 1.5–5.4; *p* = 0.001) (Table 2). After adjustment for clinical covariates (gender, time from symptom onset to PCI, infarct localization, and ischemic postconditioning), the association remained significant (OR of 2.3, 95% CI 1.1–4.6, *p* = 0.021). After further adjustment for peak CRP and troponin T, however, the association between dsDNA and infarct size was lost (Table 2).

### 3.4. Associations between NET Markers and Left Ventricular Remodeling

Day 1 dsDNA levels were significantly inversely correlated to LVEF measured both in the acute phase and after 4 months (*r* = −0.323, *p* < 0.001 and *r* = −0.285, *p* < 0.001, respectively). The same pattern was observed for dsDNA measured immediately after PCI. MPO-DNA levels did not correlate with LVEF at any time point. Neither dsDNA nor MPO-DNA correlated significantly to a change in LVEDVi or LVESVi from the acute phase to 4 months.

Based on trend analyses for quartiles of dsDNA and MPO-DNA on day 1 (Supplementary [Supplementary-material supplementary-material-1]), the median was identified as a cut-off for low and high levels as related to LVEF and a change in LVEDVi at 4-month follow-up. As outlined in Figures 2(f)–2(h), patients with high levels of dsDNA on day 1 had significantly lower LVEF acutely and after 4 months, as well as a larger change in LVEDVi (*p* ≤ 0.014 for all). No significant associations with LV remodeling indices were encountered for MPO-DNA beyond a larger change in LVEDVi at 4 months for low levels of MPO-DNA on day 1 (6.1 (-1.1, 13.8) vs. 2.2 (-4.3, 12.1) ml/m^2^, *p* = 0.025).

In univariate regression analyses, patients with high dsDNA levels on day 1 had significantly higher risk of having reduced LVEF (defined as ≤49%) at 4-month follow-up with an OR of 3.7 (95% CI 2.0–6.8, *p* < 0.001) (Table 3). After adjustment for potential covariates (gender, time from symptom onset to PCI and infarct localization), these patients were still significantly more likely to have reduced LVEF (OR 3.0, 95% CI 1.6–5.7, *p* = 0.001). After further adjustment for peak CRP and troponin T, however, the association was lost (Table 3). High dsDNA on day 1 was also significantly associated with a large change in LVEDVi (>10 ml/ml^2^) after 4 months (OR 2.1, 95% CI 1.2–3.8, *p* = 0.010), remaining statistically significant after adjustment for gender and infarct localization, but not when adjusting for peak troponin T or peak CRP (data not shown).

### 3.5. Associations between NET Markers and Adverse Clinical Events

During 12-month follow-up, 20 patients (7.3%) experienced an adverse clinical event. As previously outlined [21], these included 3 reinfarctions, 2 urgent revascularizations, 9 hospitalizations for heart failure, and 6 deaths.

Patients experiencing an adverse clinical event during follow-up had significantly higher dsDNA levels before PCI, immediately after PCI, and on day 1 and borderline significantly lower MPO-DNA levels at 4-month follow-up than patients not experiencing an adverse clinical event (Table 4).

**Figure 3:**
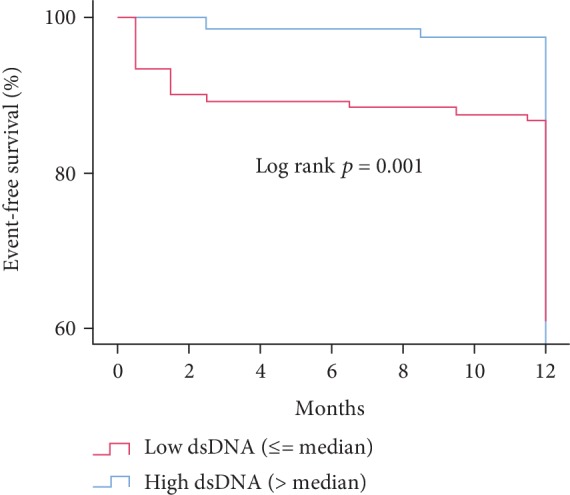
Adverse clinical events during 12-month follow-up according to dsDNA levels measured on day 1 (*n* = 251). dsDNA: double-stranded deoxyribonucleic acid. MPO-DNA: myeloperoxidase-deoxyribonucleic acid. PCI: percutaneous coronary intervention. dsDNA ≤ median: ≤366 ng/ml. dsDNA > median: >366 ng/ml. Kaplan-Meyer's survival plot.

For further survival analyses, dsDNA and MPO-DNA levels were dichotomized into low and high levels with the median as the cut-off. Patients with high dsDNA levels before PCI and on day 1 were significantly more likely to experience an adverse clinical event during follow-up than patients with low dsDNA levels (before PCI log rank *p* = 0.032, Figure 3). No such association was observed for high/low MPO-DNA levels at any time point (data not shown). In Cox's regression analyses adjusted for either peak troponin T or TIMI risk score, dsDNA levels on day 1 were still associated with an increased risk of adverse clinical endpoints (hazard ratio (HR) 5.1, 95% CI 1.4–18.4, *p* = 0.012 and HR 5.2, 95% CI 1.5–18.5, *p* = 0.009, respectively) (Table 5).

## 4. Discussion

In this substudy of the POSTEMI trial, the main findings are as follows: (1) circulating NET markers were elevated in the acute phase of STEMI; (2) high levels of dsDNA the first day after STEMI were associated with larger myocardial infarcts and adverse left ventricular remodeling; and (3) high dsDNA levels the first day after STEMI were associated with adverse clinical outcome during 12-month follow-up, and also after adjustment for the degree of myocardial necrosis assessed by peak troponin T levels. In summary, these observations indicate that extracellular nuclear material could be involved in the post-STEMI inflammatory cascade, possibly impacting clinical outcomes.

The time profile of the NET markers with high levels in the acute phase is well in line with the results from our previous smaller study, in which dsDNA was elevated until five days after STEMI [14]. From these observations, it may be suggested that NETs are most abundant in the immediate post-STEMI proinflammatory phase. The fact that PAD4 mRNA levels were not linked to NET marker levels was somewhat surprising, but could be related to the type of NETosis, as the contribution of PAD4 in vital NETosis lately has been questioned [22, 23].

High dsDNA levels the first day after STEMI were associated with more frequent MVO and greater myocardial injury, the latter measured by area at risk, infarct size, and myocardial salvage index. MVO is part of the ischemia-reperfusion (IR) injury occurring immediately after reperfusion and is caused by the occlusion of coronary microvessels by atherosclerotic debris and cells including neutrophils [24]; thus, it seems likely that neutrophil-derived NETs also participate in MVO. The association between high dsDNA levels and infarct size measured by CMR is also in line with the scarce amount of comparable existing data [14]. The fact that high dsDNA levels in the acute phase of STEMI were associated with large infarcts (>75th percentile) when adjusting for the ischemic time and infarct localization, but not peak CRP and troponin T, is worth noticing. As CRP was significantly correlated to dsDNA levels at day 1, they could both reflect the same proinflammatory milieu. Adjusting for troponin T might, however, not be justified in an explorative study like this as troponin T and infarct size are interdependent covariates. Nevertheless, the lack of association with infarct size when adjusting for troponin T renders the possibility that dsDNA measured in the peripheral circulation also reflect cardiomyocyte cell death, in addition to NETosis. The lack of correlation between PAD4 expression and NET markers in this cohort supports this. It is, however, interesting to observe that dsDNA was at its highest before revascularization (PCI), that is, prior to the usual peak of troponin T. Unfortunately, the exact time for peak troponin T was not available in this study. Whether high levels of dsDNA participate in the IR injury during revascularization, for instance through mediating MVO and thereby exacerbating cardiomyocyte cell death, troponin T release, and infarct size cannot be concluded from this observational study and warrants further investigation.

High dsDNA levels the first day after STEMI were also associated with lower LVEF and poorer left ventricular function at 4-month follow-up. The contribution of excessive or prolonged inflammation to adverse LV remodeling after STEMI is well established [3, 25], and neutrophils are suggested to play a pivotal role [5]. Experimental models have previously indicated the presence of NETs in IR-challenged myocardium based on improved LVEF in rats given a combination of tissue plasminogen activator and DNAse and higher LVEF 24 hours after coronary occlusion in PAD4-/- mice compared to wild type mice [26, 27]. Reports on NET levels in association with left ventricular remodeling after STEMI in humans have, to the best of our knowledge, not been published so far. Again, the lack of significant associations to low LVEF and adverse LV remodeling by a large change in LVEDVi when adjusting for troponin T is worth noticing, but could follow the same line of reasoning as described above.

Lastly, patients with high dsDNA levels the first day after STEMI were more likely to experience an adverse clinical outcome during 12-month follow-up. This association remained statistically significant when adjusting for myocardial necrosis by troponin T, again underpinning the potential independent contribution of dsDNA beyond being a marker of cardiomyocyte necrosis. It is interesting to note that also in patients with stable CAD [28, 29], circulating NET markers have repeatedly been observed to associate with the risk of adverse clinical outcomes, suggesting that NET effects extend beyond those related to infarct size and LV remodeling after STEMI.

### 4.1. Limitations

The observational nature of the study impedes any causal interference. Inconsistent results for the two NET markers further hamper interpretation of the data. The moderate intercorrelations between dsDNA and MPO-DNA are, nevertheless, comparable to previous reports [14, 29], emphasizing that the structure and composition of circulating NETs, as well the optimal methods for detection, have yet to be identified. Moreover, plasma may be better than serum for the detection of NET components [30]. PAD4 expression was measured in all circulating leukocytes, complicating interpretation of these results in relation to neutrophil-specific activity. Lastly and of outmost importance, interpretation of the dsDNA findings is challenging as dsDNA could be a waste product of dying cardiomyocytes, an active effector of the neutrophil-mediated inflammatory response to myocardial damage, or actually represent both processes. Although the current study cannot dissect the origin of dsDNA, it gives rise to further embark the roles of NET-associated mediators in STEMI.

### 4.2. Conclusions

In this cohort of STEMI patients, high circulating day 1 dsDNA levels after PCI were associated with myocardial infarct size, more adverse left ventricular remodeling, and poor clinical outcome during 12-month follow-up. Although the origin of dsDNA could be discussed, these observations indicate a potential role for dsDNA in acute myocardial ischemia.

## Figures and Tables

**Table 1:** Baseline characteristics of the total population and according to levels of dsDNA on day 1.

	All patients (*n* = 272)	dsDNA levels (*n* = 251)		
		<median	>median	*p*
Age (years)	60 (53, 67)	60 (53, 68)	61 (53, 67)	0.934
Male gender	223 (82)	99 (77)	109 (89)	**0.018**
Current smoker	139 (51)	60 (47)	67 (55)	0.229
Body mass index (kg/m^2^)	26.6 (24.4, 29.1)	26.8 (24.5, 29.2)	26.3 (24.3, 29.2)	0.701
Hypertension	73 (26.8)	35 (27)	35 (29)	0.844
Hypercholesterolemia	26 (9.6)	10 (8)	14 (11)	0.336
Diabetes mellitus	17 (6.3)	8 (6)	8 (7)	0.934
Time, symptoms to PCI (min)	185 (126, 265)	163 (113, 274)	198 (139, 265)	0.117
Anterior MI^1^	131 (48.2)	54 (42)	69 (56)	**0.028**
Peak troponin T (ng/l)	5865 (3302, 10337)	4918 (2463, 7186)	7760 (4702, 12984)	**<0.001**
Peak CRP (mg/l)	19 (7, 47)	12.4 (5.7, 35.5)	30.0 (11.8, 70.2)	**<0.001**
HbA1c (%)	6.0 (5.7, 6.2)	6.0 (5.7, 6.2)	6.0 (5.8, 6.2)	0.233
Admission creatinine (*μ*mol/l)	70 (62, 81)	68 (60, 80)	73 (64, 84)	**0.039**
Admission cholesterol (mmol/l)	5.2 (4.5, 6.0)	5.2 (4.7, 6.0)	5.0 (4.3, 6.0)	0.308

Values are presented as median values with 25th and 75th percentiles or proportions (%). ≤median: dsDNA levels ≤ 366 ng/ml. >median: dsDNA levels > 367 ng/ml. CRP: C-reactive protein. HbA1c: glycosylated hemoglobin. MI: myocardial infarction. PCI: percutaneous coronary intervention. ^1^Anterior vs. inferior or posterior MI.

**Table 2:** Crude and multivariable adjusted odds ratio (OR) for large final infarct size.

	OR	95% CI	*p* value
*Univariate analyses*			
High dsDNA on day 1^1^	2.9	1.5–5.4	**0.001**
Age, per year	0.99	0.96–1.02	0.400
Male gender	3.3	1.2–8.7	**0.017**
Time, symptoms to PCI (ln), per SD	1.2	0.9–1.6	0.275
Anterior MI	8.2	4.0–16.8	**<0.001**
Ischemic postconditioning	0.4	0.2–0.8	**0.007**
Total cholesterol (mmol/l), admission	0.94	0.72–1.23	0.668
Peak CRP (ln), per SD	3.6	2.3–5.6	**<0.001**
CRP after 4 months (ln), per SD	1.2	0.9–1.6	0.163
Peak troponin T (ln), per SD	21.3	9.3–48.9	**<0.001**
*Multivariable analyses*			
*Model 1*			
High dsDNA on day 1^1^	2.3	1.1–4.6	**0.021**
Male gender	2.7	0.9–7.9	0.078
Time, symptoms to PCI (ln), per SD	1.4	0.96–1.99	0.082
Anterior MI	7.3	3.4–15.6	**<0.001**
Ischemic postconditioning	0.4	0.2–0.8	**0.007**
*Model 2*			
High dsDNA on day 1^1^	1.4	0.6–3.1	0.458
Male gender	4.1	1.1–15.3	**0.037**
Time, symptoms to PCI (ln), per SD	1.4	0.9–2.2	0.095
Anterior MI	5.5	2.4–12.8	**<0.001**
Ischemic postconditioning	0.3	0.1–0.6	**0.003**
Peak CRP (ln), per SD	3.1	1.9–5.1	**<0.001**
*Model 3*			
High dsDNA on day 1^1^	0.7	0.3–1.8	0.456
Male gender	1.5	0.4–6.1	0.535
Time, symptoms to PCI (ln), per SD	1.1	0.7–1.7	0.744
Anterior MI	3.1	1.3–7.7	**0.014**
Ischemic postconditioning	0.3	0.1–0.8	**0.012**
Peak troponin T (ln), per SD	16.6	6.5–42.2	**<0.001**

Large final infarct size is defined as >75th percentile (>22.8% of LV volume) at CMR 4 months after the STEMI. ^1^>median (366 ng/ml). CI: confidence interval. CRP: C-reactive protein. dsDNA: double-stranded deoxyribonucleic acid. ln: natural logarithm. MI: myocardial infarction. PCI: percutaneous coronary intervention. SD: standard deviation.

**Table 3:** Crude and multivariable adjusted odds ratio (OR) for reduced LVEF after 4 months.

	OR	95% CI	*p* value
*Univariate analyses*			
High dsDNA levels on day 1^1^	3.7	2.0–6.8	**<0.001**
Age, per year	1.00	0.97–1.02	0.760
Male gender	3.7	1.4–9.7	**0.009**
Time, symptoms to PCI (ln), per SD	1.4	1.0–1.9	**0.026**
Anterior MI	3.6	2.0–6.5	**<0.001**
Ischemic postconditioning	0.9	0.5–1.5	0.641
Multivessel disease	0.9	0.5–1.6	0.667
Peak CRP (ln), per SD	3.1	2.1–4.6	**<0.001**
CRP after 4 months (ln), per SD	1.5	1.2–2.0	**0.006**
Peak troponin T (ln), per SD	10.5	5.6–20.0	**<0.001**
*Multivariable analyses*			
*Model 1*			
High dsDNA levels on day 1^1^	3.0	1.6–5.7	**0.001**
Male gender	2.9	1.0–8.3	**0.048**
Time, symptoms to PCI (ln), per SD	1.5	1.1–2.1	**0.022**
Anterior MI	3.0	1.6–5.8	**0.001**
*Model 2*			
High dsDNA levels on day 1^1^	2.1	0.99–4.33	0.052
Male gender	3.1	0.95–10.09	0.061
Time, symptoms to PCI (ln), per SD	1.5	1.0–2.2	**0.042**
Anterior MI	2.8	1.3–5.9	**0.008**
Peak CRP (ln), per SD	2.2	1.4–3.4	**<0.001**
CRP after 4 months (ln), per SD	1.4	0.96–1.95	0.085
*Model 3*			
High dsDNA levels on day 1^1^	1.4	0.6–3.0	0.442
Male gender	2.2	0.6–7.2	0.212
Time, symptoms to PCI (ln), per SD	1.3	0.9–1.9	0.215
Anterior MI	1.2	0.5–2.6	0.692
Peak troponin T (ln), per SD	7.9	3.9–15.8	**<0.001**

Reduced LVEF is defined as LVEF ≤ 49% measured by CMR 4 months after STEMI. ^1^>median (366 ng/ml). CI: confidence interval. CRP: C-reactive protein. dsDNA: double-stranded deoxyribonucleic acid. ln: natural logarithm. MI: myocardial infarction. PCI: percutaneous coronary intervention. SD: standard deviation.

**Table 4:** NET marker levels according to adverse clinical events after 12-month follow-up.

	Adverse clinical event -	Adverse clinical event +	*p*
dsDNA (ng/ml)			
Before PCI	450 (385, 538)	506 (415, 745)	**0.022**
After PCI	433 (380, 506)	499 (387, 630)	**0.037**
Day 1	360 (327, 406)	402 (376, 436)	**0.005**
After 4 months	338 (310, 371)	349 (318, 368)	0.833
MPO-DNA (OD)			
Before PCI	0.239 (0.205, 0.294)	0.306 (0.196, 0.359)	0.210
After PCI	0.232 (0.203, 0.272)	0.225 (0.212, 0,254)	0.454
Day 1	0.230 (0.182, 0.341)	0.274 (0.189, 0.330)	0.484
After 4 months	0.195 (0.155, 0.247)	0.158 (0.140, 0.205)	**0.045**

Adverse clinical event is defined as a composite of death, myocardial infarction (MI), unscheduled revascularization ≥ 3 months after the index infarction, stroke, or rehospitalization for heart failure. dsDNA: double-stranded deoxyribonucleic acid. MPO-DNA: myeloperoxidase-deoxyribonucleic acid. OD: optical density units. PCI: percutaneous coronary intervention.

**Table 5:** Hazard ratios for experiencing an adverse clinical event during 12-month follow-up.

	HR	95% CI	*p*
Age, per year	0.98	0.94–1.02	0.294
Male gender	0.7	0.2–1.8	0.420
Ischemic postconditioning	0.5	0.2–1.3	0.179
Time, symptoms to PCI (ln), per SD	1.1	0.7–1.7	0.737
Peak troponin T (ln), per SD	1.7	1.0–2.8	**0.037**
Peak CRP (ln), per SD	3.1	1.7–5.5	**<0.001**
Baseline NT-proBNP (ln), per SD	1.1	0.7–1.8	0.650
TIMI risk score, per point	1.3	1.1–1.6	**0.016**
*High dsDNA before PCI*	2.9	1.0–8.0	**0.043**
Adjusted for peak troponin T	2.6	0.9–7.3	0.065
Adjusted for peak CRP	2.4	0.8–6.8	0.105
Adjusted for TIMI risk score	2.7	1.0–7.6	0.057
*High dsDNA on day 1*	5.9	1.7–20.3	**0.005**
Adjusted for male gender	6.7	1.9–23.2	**0.003**
Adjusted for peak troponin T	5.1	1.4–18.4	**0.012**
Adjusted for peak CRP	3.5	1.0–12.5	0.058
Adjusted for TIMI risk score	5.2	1.5–18.1	**0.009**

High dsDNA is defined as >median (before PCI: >452 ng/ml; day 1: >367 ng/ml). Adverse clinical event is defined as a composite of all-cause mortality, myocardial infarction, unscheduled revascularization ≥ 3 months after the index infarction, rehospitalization for heart failure, or stroke. CI: confidence interval. CRP: C-reactive protein. HR: hazard ratio. PCI: percutaneous coronary intervention. SD: standard deviation. TIMI: thrombolysis in myocardial infarction.

## Data Availability

The data used to support the findings of this study are available from the corresponding author upon request.

## Supplementary Materials

Supplementary Materials CONSORT 2010 checklist. Supplementary Figure 1: study flow diagram. Supplementary Figure 2: indices of myocardial injury measured by CMR according to quartiles of dsDNA and MPO-DNA on day 1. Supplementary Figure 3: left ventricular remodeling measured by CMR according to quartiles of dsDNA and MPO-DNA on day 1.
